# Metastatic biomarkers in synovial sarcoma

**DOI:** 10.1186/s40364-017-0083-x

**Published:** 2017-02-07

**Authors:** Rosalia de Necochea-Campion, Lee M. Zuckerman, Hamid R. Mirshahidi, Shahrzad Khosrowpour, Chien-Shing Chen, Saied Mirshahidi

**Affiliations:** 1 Biospecimen Laboratory, Loma Linda University Cancer Center, Loma Linda University School of Medicine, 11175 Campus Street, Chan Shun Pavilion 11017, Loma Linda, CA 92354 USA; 2 Department of Orthopaedic Surgery, Loma Linda University Medical Center, 11406 Loma Linda Drive, Suite 218, Loma Linda, CA 92354 USA; 3 Division of Hematology/Oncology, Loma Linda University School of Medicine, 11175 Campus Street, Chan Shun Pavilion 11015, Loma Linda, CA 92354 USA; 4 Chapman University, One University Drive, Orange, CA 92866 USA

**Keywords:** SYT-SSX, CDCA2, KIF14, IGFBP7, Secernin-1, E2H2, MMPs, NY-ESO-1, CXCR4

## Abstract

Synovial sarcoma (SS) is an aggressive soft tissue sarcoma (STS) that typically occurs in the extremities near a joint. Metastatic disease is common and usually occurs in the lungs and lymph nodes. Surgical management is the mainstay of treatment with chemotherapy and radiation typically used as adjuvant treatment. Although chemotherapy has a positive impact on survival, the prognosis is poor if metastatic disease occurs. The biology of sarcoma invasion and metastasis remain poorly understood. Chromosomal translocation with fusion of the SYT and SSX genes has been described and is currently used as a diagnostic marker, although the full impact of the fusion is unknown. Multiple biomarkers have been found to be associated with SS and are currently under investigation regarding their pathways and mechanisms of action. Further research is needed in order to develop better diagnostic screening tools and understanding of tumor behavior. Development of targeted therapies that reduce metastatic events in SS, would dramatically improve patient prognosis.

## Background

Synovial sarcoma (SS) is the fourth most common type of soft tissue sarcoma (STS) and accounts for 5–10% of all soft tissue sarcomas [1]. SS was originally described by Simon in 1865, and given the name “synovial sarcoma” by Sabrazes et al. in 1934 based on a similar appearance to developing synovial tissue under light microscopy [2]. SS has a tendency to arise in the soft tissue surrounding larger joints, but it does not have a synovial cell origin [3]. While the exact cellular origin is an ongoing topic of investigation, it is likely to arise from undifferentiated mesenchymal stem cells [4]. Compared with other soft tissue sarcomas, SS occurs predominantly in younger adults with a median age of diagnosis of 35 years [5]. The most common tumor location is in the extremities, where approximately 70% of these tumors develop [6], and these patients have significantly better long term survival outcomes than those with non-extremity involvement [7]. In patients with localized disease, 10 year survival outcomes vary from 8 to 88% depending on tumor size and location [8]. Standard treatment for synovial sarcoma is tumor resection frequently accompanied by radiotherapy and/or chemotherapy, although some data suggests that therapies in addition to surgery substantially increase long-term metastatic risks [7].

Metastasis negatively impacts patient prognosis and significantly reduces survival outcomes [9]. For patients who present with or develop metastatic tumors median survival outcomes vary from 7 to 37 months depending on lymph node involvement and metastatic location [10]. SS can evolve slowly and there is a high incidence of late metastasis which occurs in about 50% of all cases [7]. Most metastatic tumors develop in the lungs (80%), although bone (9.9%) and liver (4.5%) are the next most frequent locations [9]. Synovial sarcoma can metastasize through the lymph nodes with clinically detectable disease found in 15–20% of newly diagnosed patients [11]. Histologically, synovial sarcoma is classified into four subtypes consisting of a biphasic (BPSS), monophasic fibrous or monophasic epithelial (MPSS), and poorly differentiated (PDSS, round cell) tumor cells [1]. While these histological subtypes do not seem to be associated with metastatic events [7, 12], they have been linked to survival. A study of 3756 SS patients registered in the National Cancer Data Base from 1998 to 2010 showed a significant difference in average 5-year survival numbers among patients with biphasic (65%), monophasic (56%) and undifferentiated (52%) tumors [13].

**Fig. 1:**
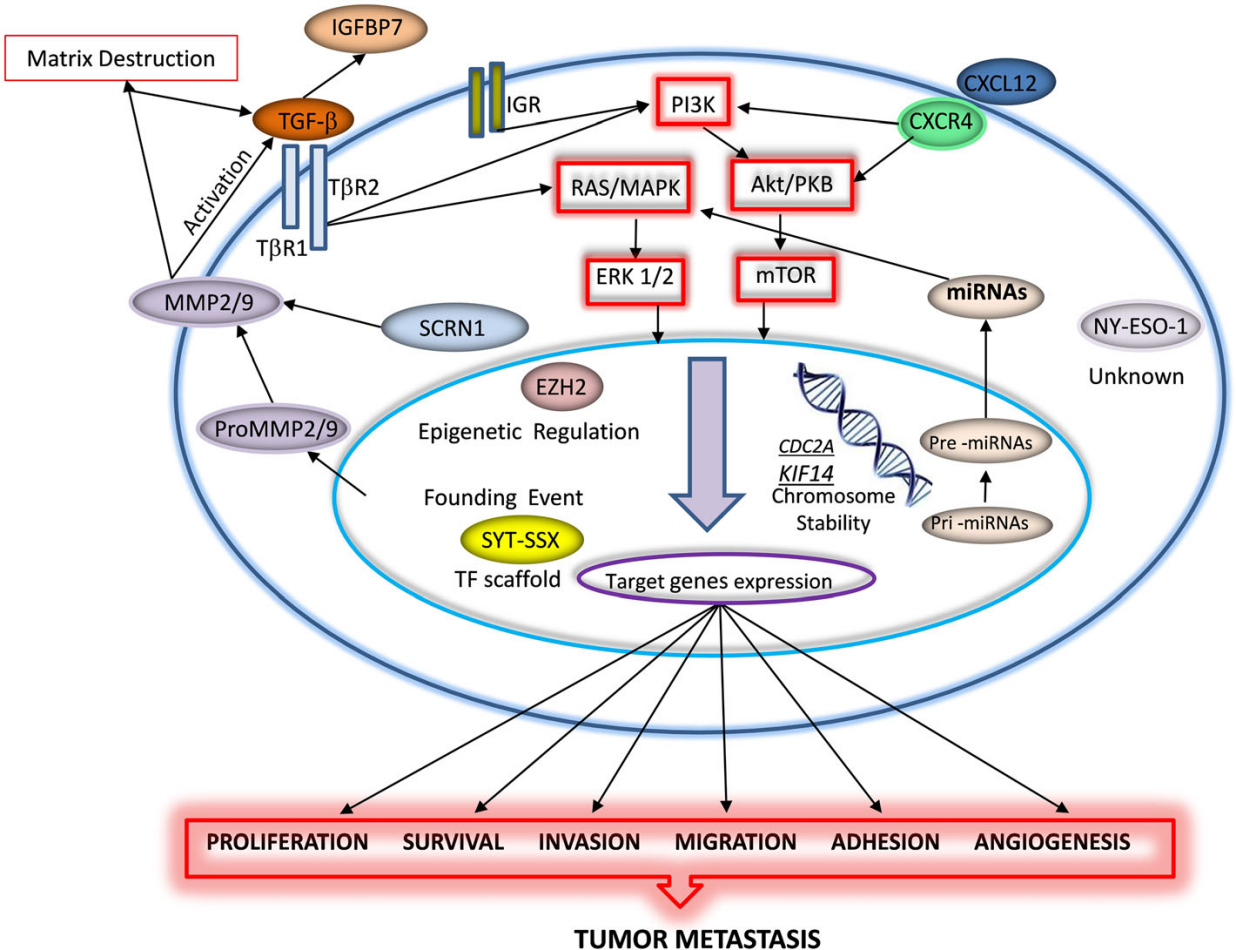
Schematic diagram summarizing functional relevance of metastatic signals in synovial sarcoma. SYT-SSX fusion is a founding event in the development of this cancer which frequently results in the production of molecular signals that promote tumor metastasis. Abbreviations: Akt (Serine/threonine kinase); CDCA2 (Cell division cycle A2); CXCR (Chemokine receptor); ERK1/2 (Extracellular signal-regulated kinase 1/2); EZH2 (Enhancer of zeste homologue 2); IGFBP7 (Insulin-like growth factor-binding protein-7); IGR (Insulin like growth factor receptor); KIF14 (Kinesin family member 14); MAPK (Mitogen-activated protein kinases); MicroRNAs (miRNAs); MMPs (Matrix metalloproteinases); mTOR (Mammalian target of rapamycin); NY-ESO-1 (New York esophageal squamous cell carcinoma 1); PI3K (Phosphatidylinositol-3-kinase); pri-miRNA (primary microRNA); pre-miRNA (precursor microRNA); PKB (Protein kinase B); RAS (Ras GTPase); SCRN1 (Secernin-1); TGF-β (Transforming growth factor beta); TβR (TGF-beta receptor); TF (transcription factor)

While many factors that influence synovial sarcoma patient outcome have been identified, tumor behavior remains highly unpredictable [14]. An extremely high level of metastasis means that further studies are needed to characterize the mechanisms that influence tumor action. Ultimately, the identification of highly relevant molecular biomarkers associated with metastatic outcomes and patient prognosis establishes the foundation for development of better therapeutic strategies to target these oncogenic factors. Several metastatic biomarkers that have been described to date are described in the following sections and summarized in Fig. 1.

## Significance of the syt-ssx fusion gene

The chromosomal translocation t(X;18)(p11.2;q11.2) fuses the SS18 (SYT) gene to the SSX gene (predominantly SSX1 or SSX2) and is regarded as a founding event in the oncogenic development of synovial sarcoma [15]. Yet, the exact transformative event of the chimeric SYT-SSX gene product has not been fully elucidated. It has been shown that SYT-SSX can interfere with assembly of BAF (BRG-/BRM-associated factor) complexes affecting the integration of a tumor suppressor component and consequent SRY (sex-determining region Y)-box 2 (SOX2) activation which stimulates cell proliferation [16]. Over 95% of SS can be characterized by expression of the SYT-SSX gene and it is used as a routine diagnostic marker for this type of cancer [15]. Although both SYT and SSX proteins contain transcriptional regulation domains, they lack any DNA binding regions, so their regulatory effects are surmised to occur through interactions with other proteins [17]. Accordingly, the SYT-SSX fusion oncogene has been shown to interact with several major epigenetic regulators as well as other DNA binding proteins and exert both direct and indirect effects on transcript regulation [17, 18]. Of note, SYT-SSX has been shown to act as a scaffold linking two master transcription regulators TLE1 (transducin like enhancer of split 1) and ATF2 (activating transcription factor 2) such that TLE1 acts as a repressor of ATF2 target genes regulating cell cycle, apoptosis, and more, demonstrating that the SYT-SSX/TLE1/ATF2 complex is important not only to oncogenic transformation but also tumor cell survival [19]. TLE has emerged as a useful diagnostic marker of SS with robust expression detected in approximately 95% of these tumors however a low level of positive staining in non-SS tumors indicates that this antibody should only be used in a panel with other antibodies to confirm diagnosis [20]. Recently, a highly specific assay to detect the association of SYT-SSX and TLE in either localized or metastatic SS tumors was developed and shown to be useful in drug discovery assays seeking to disrupt this interaction [21].

A number of studies have investigated the association between SYT-SSX gene fusion type and metastatic risk. Some have reported that the SYT-SSX1 fusion approximately doubles the risk of metastatic tumor development compared with SYT-SSX2 [22, 23]. Intriguingly, a similar pattern was evident in a survival study demonstrating a median survival of 6.1 years for SS patients with an SYT-SSX1 fusion gene compared with 13.7 years for those with SYT-SSX2 [24]. In contrast, several other studies report no significant associations between SYT-SSX fusion type and metastasis or survival [7, 25]. These conflicting data appear to suggest that the influence of unspecified additional factors have a much larger role in determining metastatic outcomes. These clinicopathological characteristics must be better defined to understand in which context SYT-SSX has an important versus an inconsequential role.

## Circulating biomarkers

Development of a diagnostic blood test to detect SS and assess metastatic risk would be a valuable tool for the management of this disease. A whole blood microRNA signature shows that SS patients demonstrate significant upregulation of seven microRNAs (miR-99a-5p, miR-146b-5p, miR-148b-3p, miR-195-5p, miR-223-3p, miR-500b-3p and miR-505-3p) compared with patients in remission and healthy controls [26]. Furthermore, expression levels of these seven microRNAs are significantly reduced by 5–50 fold increments after local tumor resection, but once again dramatically upregulated if a patient develops recurrent local or metastatic disease [26]. Thus, since a clearly defined microRNA expression panel can be used to distinguish SS from other malignancies as well as characterize metastatic and recurrent tumor status, this method could be useful in therapeutic monitoring of patients [26]. miRNAs have a critical role in activation of the Ras GTPase/Mitogen-activated protein kinases (Ras/MAPK) pathway necessary for tumor development [27], and miRNA suppression significantly inhibits SS cell proliferation in vitro [28].

Detection of a circulating SYT-SSX fusion gene product could be another important method for assessing metastatic risk in patients with SS. In culture, it has been shown that SS cells produce microvesicles containing the SYT-SSX fusion gene, yet this research group was unable to detect the biomarker in microvesicles or PBMCs (peripheral blood mononuclear cell) obtained from patient blood samples regardless of their metastatic status [29]. In contrast, the SYT-SSX gene was detected in peripheral blood in a case study of a single SS patient who developed multiple lung metastasis 2 months after local tumor resection from the thigh [30]. The presence of circulating tumor cells (CTC) could be indicative of a patient’s metastatic risk given their potential to extravasate and form tumors in new locations [31], and CTC abundance may account for discrepancies in SYT-SSX detection abilities. CTCs can be readily isolated from patient blood by size exclusion, and CTC quantities vary considerably among different SS patients [32].

## Cinsarc signature genes

Gene expression profiling of soft tissue sarcomas revealed a prognostic panel of 67 genes (CINSARC, complexity index in sarcomas) with functional roles in mitosis and chromosome management that are also highly predictive of metastatic risk [33]. While many CINSARC genes have been identified as molecular markers associated with metastasis in other types of cancers, they are usually ascribed a proliferative function, although they may actually have a greater role in chromosome instability [33]. When the CINSARC classification criteria were used to stratify SS tumor specimens into two prognostic groups, a highly significant difference was observed in metastatic outcomes among these patients [34]. However, since the CINSARC profile emerged from an analysis of multiple types of soft tissue sarcomas, these authors sought to determine if a better metastatic prognostic profile could be developed for SS [34]. Comparison of whole genome expression in 51 metastatic and 49 non-metastatic SS tumors revealed significant upregulation of 59 genes in metastatic specimens, 24% of which were common to the CINSARC classification panel [34]. Importantly, singular expression of the 2 most differentially regulated genes, CDCA2 (cell division cycle A2) and KIF14 (kinesin family member 14), could better predict metastatic outcomes than the overall CINSARC score in this patient cohort [34]. Functionally, both of these genes code for proteins that help to maintain chromosome integrity and are essential for the completion of cytokinesis. KIF14 is localizes to the central spindle during late phase mitosis and its inhibition in tumor cells results in cell cycle arrest and the formation of binucleated cells [35]. KIF14 was also found to regulate adhesive components on the tumor cell surface influencing migratory and invasive properties that promote cell motility during metastasis [36]. CDCA2 has critical catalytic and structural functions during late mitosis that help coordinate chromosome segregation and nuclear envelope reformation after division of nuclear contents [37].

## IGFBP7

Insulin-like growth factor-binding protein-7 (IGFBP7,) also termed IGFBP-related protein-1 (IGFBP-rP1), is a secreted 31-kDa protein belonging to the IGFBP family [38]. In various cancer types including hepatocellular carcinoma [39], breast [40], brain [41], and colon [42], IGFBP7 can function as a tumor suppressor and have the ability to suppress proliferation, adhesion, angiogenesis, survival, or induce apoptosis and senescence [43–45]. Yet the role of this protein in tumor behavior is complex, as it undergoes extensive proteolytic processing that reverses its cellular function and influence over cell proliferation and adhesion activities [46]. In the tumor stroma microenvironment, IGFBP7 expression is closely related with the transforming growth factor beta (TGF-β secretion [47], which is a potent factor promoting tumor cell invasiveness and metastasis [48, 49]. TGF-β upregulates expression of IGFBP7 and angiogenic capacity of tumor cells [50]. Recently, it was shown that IGFBP7 expression in SS was higher that other types of STSs and significantly associated with metastatic events [51]. In addition, nuclear expression of another IGFBP family protein, insulin like growth factor 1 receptor (IGF1R),, was significantly related to poor survival in SS patients who did not receive adjuvant chemotherapy [52]. In sarcoma, IGF1R activation is known to activate the phosphatidylinositol-3-kinase/serine/threonine kinase Akt/Mammalian target of rapamycin (PI3K/Akt/mTOR) pathway which promotes cancer progression and metastasis [53]. These studies, highlight the potential for proteins from the IGF family to be used as prognostic biomarkers to help guide treatment decisions in SS.

## MMPs

The matrix metalloproteinases (MMPs), a family of zinc and calcium dependent proteolytic enzymes, are involved in the degradation extracellular matrix (ECM) components and play key roles in tumor cell invasion and metastasis [54]. Notably, the proteolytic activity of MMPs can help release inactive TGFB in the extracellular space so that it can bind to its receptors and activate downstream pathways such as PI3k/Akt and MAPK which are critical to the epithelial mesenchymal transition (EMT) process underlying metastasis [55]. Benassi et al. demonstrated that high levels of MMP2 and MMP9 in biopsied tissue from patients with SS, was significantly associated with metastasis (*P* = 0.008 and *P* = 0.005, respectively) [56]. In addition, lack of expression the tissue inhibitors of metalloproteinases (TIMP) was found to be a poor prognostic factor for disease-free survival in synovial sarcoma (*P* = 0.009) [56]. The proteolytic activity of MMPs and their activation process can be inhibited by the TIMPs [57]. The presence of TIMPs can suppress metastasis by preserving ECM integrity [58]. It has been shown that a decrease in TIMP-1 correlated with a poor outcome in high-risk STS [59] and colorectal cancer patients [60]. Both MMPs and TIMPs can further be evaluated as biological markers for predicting progression, metastasis and prognosis of human SS.

## Secernin-1

Secernin-1 (SCRN1), a 50-kDa cytosolic protein, is a member of the secernin gene family that regulates exocytosis in mast cells through a mechanism that has not been well defined [61]. Exocytosis is a process by which cells transport and release secretory products through the cytoplasm to the cell membrane and several studies have described that this promotes tumor growth, metastasis and invasion [62, 63]. In colon cancer, SCRN1 expression promoted exocytosis secretion of MMP2 and MMP, while silencing this gene reduced MMP2 secretion, inhibited cell proliferation and decreased invasion capability [63]. The poor prognostic significance of SCRN1 expression in colon cancer [63, 64], is contrary to that reported in synovial sarcoma [65]. A proteomics analysis of tumor specimens collected from 13 SS patients, identified SCRN1 as positive prognostic factor with significantly higher expression among patients who were alive and disease free for at least 5 years [65]. Further analysis of SCRN1 expression in 45 SS tumor specimens revealed a 5-year survival rate was 77.6 and 21.8% for patients with secernin-1 positive and negative primary tumors, respectively (*p* = 0.0015), and significantly associated with metastatic outcomes [65]. Metastasis-free survival was significantly higher (62.8% vs. 16.7%) in the SS patient group with SCRN1 positive tumors compared to that with SCRN1 negative tumors (*p* = 0.0012) [65].

## EZH2

Enhancer of zeste homologue 2 (EZH2) is a member of the polycomb group (PcG) protein family, which is composed of epigenetic transcriptional regulators that participate in cell cycle regulation, DNA damage repair, cell differentiation, senescence, and apoptosis [66]. In cancer, EZH2 expression is associated with a worse prognosis and required for promotion of metastasis [67]. In SS, overexpression of EZH2 helps to distinguish PDSS, which is defined by high cellularity, high nuclear grade, high mitotic activity and an agresssive clinical course that tends to include early recurrence and metastasis, from the MPSS and BPSS histological subtypes [68]. EZH2 overexpression in SS is correlated with high H3K27 trimethylation, which facilitates chromatin compaction and gene silencing [68]. Importantly, high expression of EZH2 is predictive of developing distant metastasis even in the better-differentiated MPSS and BPSS subtypes [68]. In a recent preclinical study, the anti-tumor effect of EZH2 inhibition was evidenced in human SS models in vitro*,* as well as xenograft and patient-derived xenograft (PDX) models in vivo [69]*.* Moreover, Ramagila et al. found high EZH2 expression to be correlated with metastatic disease in pediatric soft tissue sarcomas [70]. Low expression of EZH2 restricts cell proliferation and induces cell cycle arrest at the G2 phase, whereas the overexpression of EZH2 can shorten the G1 phase of the cell cycle and lead to cell accumulation in the S phase [71, 72]. Moore et al. showed that EZH2 knockdown is sufficient to reduce distant metastasis in vivo [73]. EZH2-specific inhibition is an active area of researcher, with several human phase 1 and 2 trials now underway, such as an ongoing phase II, multicenter study of the EZH2 inhibitor tazemetostat in adult subjects with INI1-negative tumors or relapsed/refractory SS (https://clinicaltrials.gov/ct2/show/NCT02601950 **)**.

## NY-ESO-1

New York esophageal squamous cell carcinoma 1 (NY-ESO-1), encoded by the *CTAG1B* gene, is a 22 kD hydrophobic protein cancer-testis antigen [74]. NY-ESO-1 is expressed in many cancers, associated with poor prognosis, and elevated metastatic risk [75]. The function of NY-ESO-1 is still unknown, but is of particular interest to researchers because it is highly immunogenic eliciting both cellular and humoral responses, and a large number of major histocompatibility complex (MHC) class I- and class II-restricted NY-ESO-1 epitopes have been identified [76]. Furthermore, NY-ESO-1 protein expression is significantly higher in metastatic versus primary tumors [75, 77, 78]. NY-ESO-1 is an attractive target for SS treatment because chemotherapy has a limited durable efficacy in relapsed or metastatic SS demonstrating the need for novel more effective therapies. NY-ESO-1 is expressed in approximately 80% of patients with SS which could be useful for distinguishing this cancer from other types of mesenchymal tumors, and identifying patients who would benefit from NY-ESO-1 targeted therapies [79]. In a clinical trial using genetically engineered T cells reactive with NY-ESO-1 in patients with metastatic synovial cell sarcoma, tumor regression was achieved in 67% of the patients [80]. Several clinical trials employing NY-ESO-1 are currently under way in patients with SS (https://clinicaltrials.gov/ct2/show/NCT01343043, NCT02609984).

## CXCR4

Chemokine receptor 4 (CXCR4), is a 352-amino acid seven-transmembrane G protein-coupled cell surface receptor with an important role in homing of hematopoietic stem cells and lymphocyte trafficking that has been found to promote cell migration, invasion and angiogenesis [52, 81]. The CXCR4 pathway has been shown to be associated with tumor progression and poor prognosis in many types of cancer including breast [82], lung [83], colon [84], melanoma [85] and soft tissue sarcomas [86]. CXCR4 promotes metastasis by activating activating extracellular signal-regulated kinase 1/2 (ERK1/2), Akt/PKB and Nuclear factor-κB (NF-κB), which increases the adhesion and invasive ability of cancer cells in part by the activity of MMP2 and MMP9 [87–89]. CXCR4 has a pivotal role in the migration of cancer cells between the primary and the metastatic site in synovial sarcoma [52, 90]. Tumor cells expressing CXCR4 that detach from the primary tumor and enter the circulatory system can migrate toward organs that express its ligand CXCL12 [91]. Lung, lymph node, bone marrow, and liver, the most frequent metastatic locations in SS [9], all express very high levels of CXCL12 [92]. A study of SS patients found that 5-year overall survival (OS) rates were 47% for those with positive CXCR4 staining, and 86% (*P* = 0.0003) for those with negative CXCR4 staining [52]. A second study, reported that 5 year survival outcomes for SS patients with positive CXCR4 staining was less than 30% [4]. Importantly, it was found that SS cultures contain a subpopulation of cells expressing high levels of CXCR4 that also express high levels stem cell markers (NANOG, OCT4, SOX2), and these cells have an increased tumor initiating capacity in xenographic mouse models [4]. Although no group has directly measured the metastatic risk of CXCRX4 expression in SS, perhaps due to limited patient numbers, expression of this marker appears to be a key factor both for cell migration and tumor propagation at a distal site. Perhaps the use of CXCR4 antagonists, many of which are already in various stages of clinical development [93], and shown to significantly reduce lung metastasis in mouse models of osteosarcoma [94], would be a beneficial treatment option for patients with metastatic SS, particularly in cases where tumors are unresectable.

## Conclusions

SS is a rare, aggressive subtype of soft tissue sarcoma. It has a predilection for metastases to multiple organs, including the lungs, lymph nodes, and bone. The ability of this tumor to metastasize to multiple organs demonstrates that the tumor can interact and invade multiple environments. The overall prognosis is poor given the high rate of metastatic disease and lack of effective therapeutic agents. Nearly all mortality in patients with SS is caused by metastatic disease, yet the biological cause of these events has not been well characterized. The full transformation that occurs with the X;18 chromosomal translocation has not been completely elucidated at this time. Several oncogenic biomarkers have been found to be elevated in SS. As summarized in Fig. 1, many of these biomarkers may be used to evaluate for recurrence and metastatic disease and also help determine prognosis. The lack of the full understanding of how the translocation and elevated biomarkers interact with the host is a significant limitation in our ability to effectively treat SS. Further research should be done to help develop a greater understanding of these interactions and the downstream effects that occur in SS, with an emphasis on preventing metastatic disease. This will not only enable patients to be monitored for progression of disease and allow for counseling regarding prognosis, but also be used to develop better treatments for this subtype of soft tissue sarcoma.

## Abbreviations

Akt: Serine/threonine kinase
ATF2: Activating transcription factor 2
BAF: BRG-/BRM-associated factor
BPSS: Biphasic synovial sarcoma
CDCA2: Cell division cycle A2
CTC: Circulating tumor cells
C-X-C motif: Chemokine
CXCL12: ligand 12
CXCR4: Chemokine receptor 4
ECM: extracellular matrix
EMT: Epithelial mesenchymal transition
ERK1/2: extracellular signal-regulated kinase 1/2
EZH2: Enhancer of zeste homologue 2
IGF1R: Insulin like growth factor 1 receptor
IGFBP7: Insulin-like growth factor-binding protein-7
IGFBP-rP1: IGFBP-related protein-1
KIF14: Kinesin family member 14
MAPK: Mitogen-activated protein kinases
MMPs: Matrix metalloproteinases
MPSS: Monophasic synovial sarcoma
mTOR: Mammalian target of rapamycin
NF-κB: Nuclear factor-κB
NY-ESO-1: New York esophageal squamous cell carcinoma 1
PBMCs: Peripheral blood mononuclear cell
PcG: Polycomb group
PDSS: Poorly differentiated synovial sarcoma
PDX: Patient-derived xenograft
PI3K: Phosphatidylinositol-3-kinase
PKB: Protein kinase B
RAS: Ras GTPase
SCRN1: Secernin-1
SOX2: SRY (sex-determining region Y)-box 2
SS: Synovial sarcoma
STS: Soft tissue sarcoma
TGF-β: Transforming growth factor beta
TIMP: Tissue inhibitors of metalloproteinases
TLE1: Transducin like enhancer of split 1
